# Chromosome-level genome assembly of humpback grouper using PacBio HiFi reads and Hi-C technologies

**DOI:** 10.1038/s41597-023-02907-4

**Published:** 2024-01-09

**Authors:** Jinxiang Liu, Huibang Sun, Lei Tang, Yujue Wang, Zhigang Wang, Yunxiang Mao, Hai Huang, Quanqi Zhang

**Affiliations:** 1 MOE Key Laboratory of Marine Genetics and Breeding, College of Marine Life Sciences/Key Laboratory of Tropical Aquatic Germplasm of Hainan Province, Sanya Oceanographic Institution, Ocean University of China, Qingdao/Sanya, China; 2 Laboratory for Marine Fisheries Science and Food Production Processes, Qingdao National Laboratory for Marine Science and Technology, Qingdao, China; 3 Hainan Yazhou Bay Seed Laboratory, Sanya, China; 4 MOE Key Laboratory of Utilization and Conservation for Tropical Marine Bioresources, Hainan Tropical Ocean University, Sanya, China

**Keywords:** Genetics, Evolution

## Abstract

The humpback grouper (*Cromileptes altivelis*), a medium-sized coral reef teleost, is a naturally rare species distributed in the tropical waters of the Indian and Pacific Oceans. It has high market value, but artificial reproduction and breeding remain limited and need to be improved. Here, we assembled the genome with 1.08 Gb, with a contig N50 of 43.78 Mb. A total of 96.59% of the assembly anchored to 24 pseudochromosomes using Hi-C technology. It contained 24,442 protein-coding sequences, of which 99.3% were functionally annotated. The completeness of the assembly was estimated to be 97.3% using BUSCO. The phylogenomic analysis suggested that humpback grouper should be classified into the genus *Epinephelus* rather than *Cromileptes*. The comparative genomic analysis revealed that the gene families related to circadian entrainment were significantly expanded. The high-quality reference genome provides useful genomic tools for exploiting the genomic resource of humpback grouper and supports the functional genomic study of this species in the future.

## Background & Summary

Groupers, as a series of important commercial and ecological reef fish, are distributed in tropical and subtropical waters worldwide. On present understanding, groupers consist of 165 species in 16 genera and vary considerably in terms of lifestyle, growth rate, and body appearance^1^. The humpback grouper is a naturally rare species that is widely distributed in the tropical waters of the Indian and Pacific Oceans^2^. The term “humpback grouper” is because its body is relatively higher than its head, which gives a humpback aspect. The humpback grouper is a medium-sized fish, which grows up to 70 cm. As a protogynous hermaphroditic species, all humpback grouper individuals are born female and can transform into male when they grow up and experience 2–5 spawning seasons. This fish has high market value and is exceedingly favored by consumers due to their high nutritional value, tasty flesh, and beautiful appearance. In recent years, overfishing has led to a sharp decrease in the wild humpback grouper population, whereas the market demand has increased rapidly. Its relatively slow growth rate, unique sex-change strategy, and susceptibility to various pathogenic diseases during cultivation severely restrict the development of artificial culture. Previous studies of humpback grouper focused on immunology, the establishment of cell lines, classification, and feed supplement^3–6^. The decoding of a high-quality reference genome could support more information on molecular biology, genetics, breeding, and conservation biology.

Recently, several types of grouper genomes have been assembled, such as giant grouper (*Epinephelus lanceolatus*), leopard coral grouper (*Plectropomus leopardus*), and red-spotted grouper (*Epinephelus akaara*)^7–9^. Traditionally, grouper identification was primarily dependent on the surface profile and phenotype. Actually, it could cause errors and challenges in taxonomy. The groupers had a close relationship in evolution. To better understand the evolutionary relationship and taxonomy, it was necessary to acquire a specific solution by molecular biology. Besides, a high-quality reference genome resource could also provide an effective tool for genetic improvement and germplasm conservation. At present, the long-read and short-read sequencing technologies have been applied to the assembled genome. It was able to obtain highly integrated genome assemblies, especially circular consensus sequencing (CCS) improved the accuracy of PacBio SMRT sequencing. The HiFi sequence updated the genome assembly between read length and base quality significantly.

In 2021, a humpback grouper genome was constructed with the assembly of 1.013 Gb (contig N50 of 18.09 Mb)^10^. In this study, we represent a chromosome-scale genome assembly and annotation of humpback grouper with the PacBio HiFi and Hi-C sequencing technologies. Approximately 1.08 Gb genome was assembled with the contig N50 43.78 Mb. BUSCO analysis showed that 97.3% of the final assembly was complete BUSCOs. Overall, this high-quality reference genome provides a valuable basis for further genetic improvement and understanding the functional genes and molecular mechanisms in humpback grouper

## Methods

### DNA sample collection, library construction, and sequencing

A female humpback grouper was collected from Hainan Chenhai Aquatic Co., Ltd. The muscle tissue was collected for DNA extraction and library construction. Genomic DNA was extracted by the QIAamp DNA purification kit (Qiagen, USA). The short fragment library was generated using the Truseq Nano DNA HT Sample Preparation Kit (Illumina, USA) with an insert size of 350 bp and the Illumina NovaSeq 6000 platform. For the HiFi read generation, DNA fragment > 30 kb was selected using BluePippin Systerm (Sage Science, USA). The library was generated using the SMRTbell Template PrepKit 2.0 (PacBio, USA), and the library was sequenced in CCS on the PacBio Sequel II platform. The Hi-C library was constructed following the standard protocol described previously with certain modifications^11^, and it was sequenced using the Illumina NovaSeq 6000 platform. A total of 53.1 Gb of Illumina data, 21.5 Gb PacBio of PacBio data, and 96 Gb of Hi-C data after trimming the low-quality reads and adaptor sequences from the raw data.

### RNA sample collection, library construction, and sequencing

The samples of eight embryonic development stages (one cell, morula, high blastula, low blastula, gastrula, somite, neurula, and before the hatching stage) were collected for RNA extraction using TRIzol reagent (Invitrogen, USA). RNA-seq libraries were constructed using Illumina TruSeq Stranded mRNA Library Prep Kit (Illumina, USA) and sequenced by the Illumina NovaSeq 6000 platform. Further, RNA extracted from embryonic samples was mixed for Iso-seq. The Iso-seq library was constructed and sequenced on the PacBio Sequel II platform. The clean data was obtained by removing reads containing adapters, reads containing poly-N and low-quality reads from the raw data. Around 55.6 Gb of RNA-seq data and 69.1 Gb of Iso-seq data were generated for genome annotation.

### Genome assembly and quality assessment

**Fig. 1:**
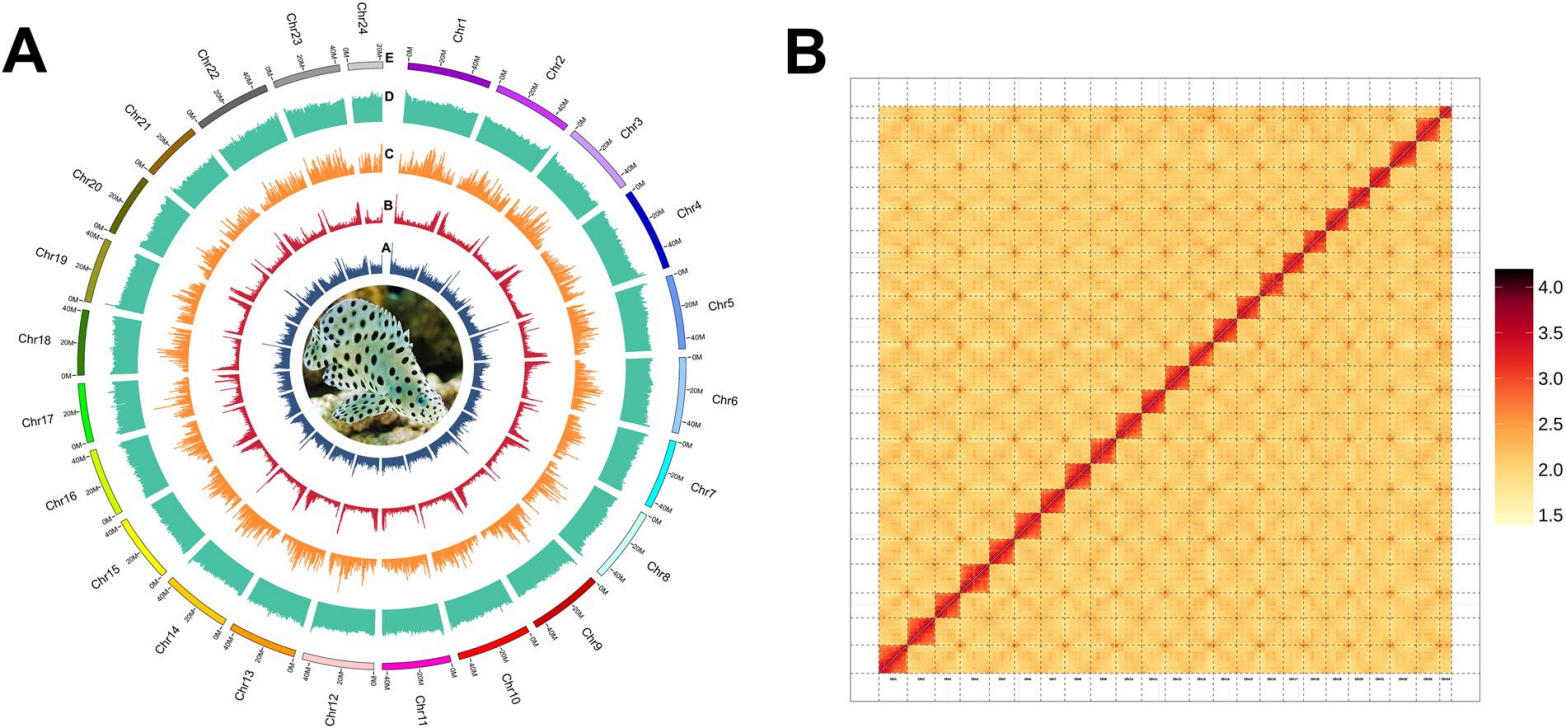
Genome assembly of the humpback grouper. (**A**) Genomic features. From inner to outer tracks: A, distribution of DNA TEs across the genome; B, distribution of RNA TEs across the genome; C, gene density across the genome; D, GC content across the genome. E, humpback grouper chromosomes. (**B**) Hi-C contact map of the humpback grouper genome. The blocks represent the contacts between one location and another. The color illustrates the contact density from red (high) to low (orange).

The characterization of the genome was estimated using the Illumina short-read data, and the 17 bp k-mer analysis was applied for estimation. The estimated genome size was 1,091.59 Mb, the heterozygosity rate was approximately 0.19%, and the repeated content was 45.81%. The genome was assembled using SOAPdenovo2 with k-mer set at 41 bp^12^. The gaps were filled with GapCloser. Then, the draft genome was corrected and re-assembled using HiFi long reads by Hifiasm 0.12-r304 with the parameters “-t 30 -D 10”^13^. The genome assembly was 1.08 Gb, with a contig N50 size of 43.78 Mb (Fig. 1A). To obtain the chromosome-level genome, we applied ALLHiC pipeline to link the mapped contigs to 24 pseudochromosomes^14^. Finally, 96.59% of scaffolds were mapped to 24 chromosomes (Fig. 1B).

To evaluate the assembled genome, BUSCO was applied to evaluate the completeness of genome assembly. A total of 3,345 BUSCO genes were identified, with 3,263 complete genes, 3,230 single-copy genes, 33 multi-copy genes, 47 fragmented genes, and 44 missing genes accounting for 97.3%, 96.3%, 1.0%, 1.4%, and 1.3% of the whole genome, respectively (Table 1).

**Table 1:** BUSCO evaluation result of humpback grouper genome.

Genome evaluation	Gene number	Percentage %
Complete BUSCOs	3,263	97.3
Complete and single-copy BUSCOs	3,230	96.3
Complete Duplicated BUSCOs	33	1.0
Fragmented BUSCOs	47	1.4
Missing BUSCOs	44	1.3
Total BUSCO groups searched	3,345	100

### Repeat and noncoding RNA annotation

**Fig. 2:**
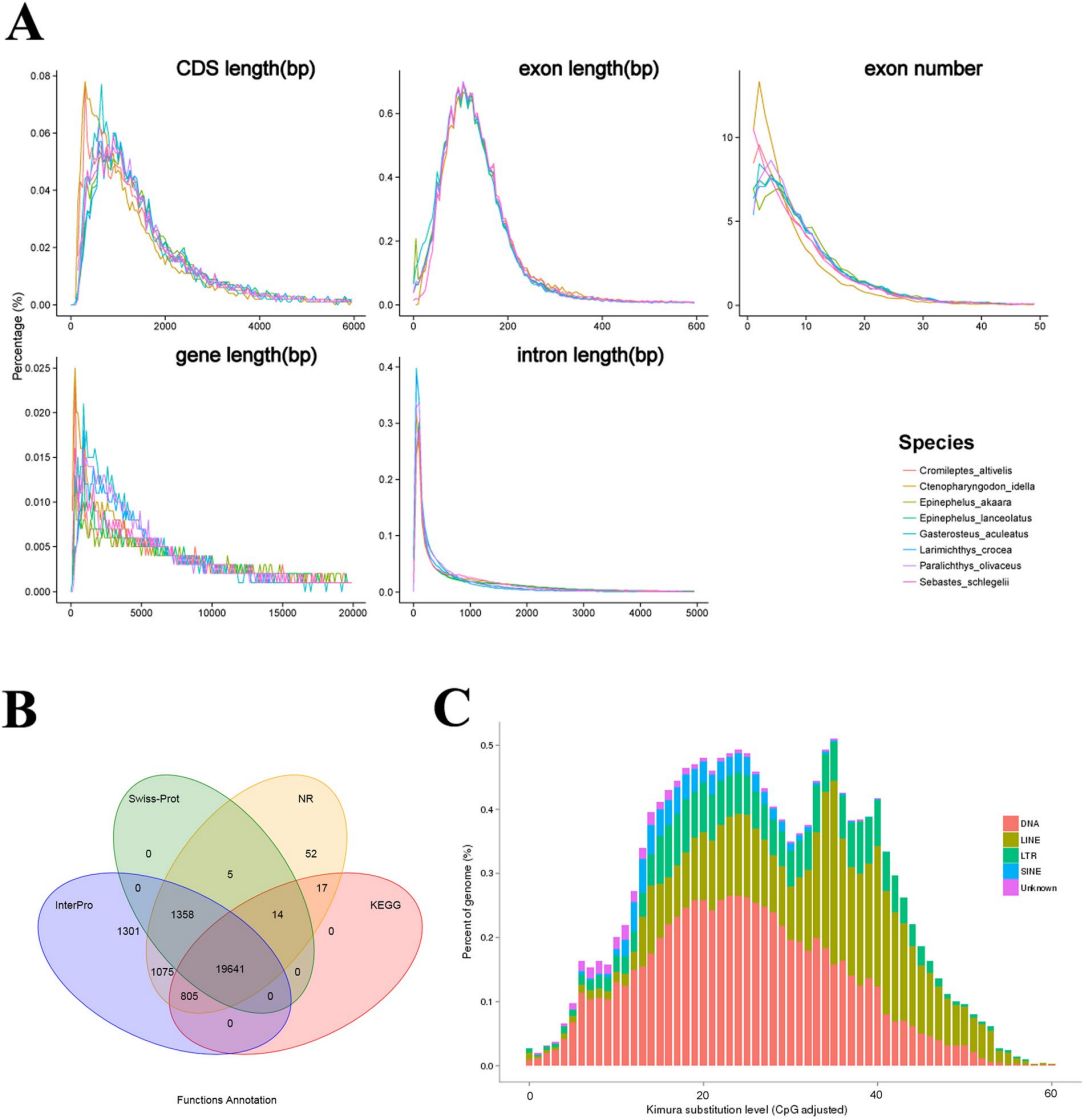
The structural and functional annotation of humpback grouper. (**A**) Comparisons of the predicted gene models between the humpback grouper genome and other teleosts, including CDS length, exon length, exon number, gene length, and intron length. (**B**) The functional annotation of humpback grouper using different databases. (**C**) The percentage of different types of repetitive elements in the humpback grouper genome.

Repeat sequences of the humpback grouper genome were identified using a combination of homology-based and *de novo* approaches. For the ab initio method, the RepeatModeler (v2.0.1)^15^, RepeatScout (v1.0.5)^16^, and LTR_finder (v1.0.6)^17^ were used to build the humpback grouper custom repeat database. In the homology-based method, the Repbase database^18^ was used to identify repeats with the RepeatMasker and RepeatProteinMask. The total length of the repetitive elements accounted for 44.38% of the humpback grouper genome (Fig. 2C). DNA transposons represented the most abundant class of repeats (17.85% of the genome) followed by long interspersed elements (LINEs, 15.20%), long terminal repeats (LTRs, 5.38%), and short interspersed elements (SINEs, 1.11%) (Table 2).

**Table 2:** Statistic results of different types of annotated repeat content.

Type	Denovo + Repbase		TE proteins		Combined TEs	
	Length (bp)	% in genome	Length (bp)	% in genome	Length (bp)	% in genome
DNA	185,549,534	17.13	17,809,686	1.64	193,277,492	17.85
LINE	164,630,235	15.20	45,118,481	4.17	184,303,613	17.02
SINE	12,052,908	1.11	0	0	12,052,908	1.11
LTR	83,542,399	7.71	7,526,000	0.69	86,240,213	7.96
Other	0	0	0	0	0	0
Unknow	22,465,884	2.07	0	0	22,465,884	2.07
Total	451,213,734	41.66	70,355,897	6.50	468,059,310	43.22

Noncoding RNAs, including rRNAs, snRNAs, miRNAs, and tRNAs, were identified by adopting INFERNAL (v1.1.2) through the Rfam database (release 13.0) for the humpback grouper genome using BLASTN (*E*-value ≤ 1e−5)^19–21^. Transfer RNA was predicted using tRNAscan (v1.3.1)^22^ with default parameters for eukaryotes. Ribosome RNAs and their subunits were predicted using the RNAmmer (v1.2)^23^. For non-coding RNA annotation, a total of 1,905 miRNA, 2,107 tRNA, 3,360 rRNA, and 1,637 snRNA were identified (Table 3).

**Table 3:** Summary statistics of noncoding RNA.

Type	Copy	Average length (bp)	Total length (bp)	% of genome
miRNA	1,905	110.68	210,842	0.019468
tRNA	2,107	75.32	158,693	0.014653
rRNA	1,680	173.81	291,993	0.026960
18 S	153	384.06	58,761	0.005426
28 S	329	307	101,003	0.009326
5.8 S	26	152.50	3,965	0.000366
5 S	1,172	109.44	128,264	0.011843
snRNA	846	149.29	126,296	0.011661
CD-box	149	114.12	17,004	0.001570
HACA-box	88	151.48	13,330	0.001231
splicing	554	155.32	86,050	0.007945

### Gene prediction and annotation

Firstly, three strategies were used for gene structure prediction, including *de novo* prediction, homology-based, and RNA-seq data-based prediction. Augustus (v2.5.5)^24^, Glimm erHMM (v3.01)^25^, SNAP^26^, Geneid^27^, and Genescan^28^, were used for *de novo* gene prediction with default settings. Protein sequences of giant grouper, black rockfish (*Sebastes schlegelii*), stickleback (*Gasterosteus aculeatus*), large yellow croaker (*Larimichthys crocea*), grass carp (*Ctenopharyngodon idella*), Japanese flounder (*Paralichthys olivaceus*), and red-spotted grouper were downloaded from Ensembl and NCBI databases. These sequences were aligned to the humpback grouper genome with TBLASTN (*E*-value ≤ 10−5), and homologous genome sequences were then aligned against matching proteins by GeneWise (v2.4.0)^29^ to generate a gene structure based on the alignment. Furthermore, the RNA-seq data from different embryonic development stages were assembled using Trinity (v2.1.1)^30^ and mapped to the humpback grouper genome by using the Cufflinks (v2.1.1)^31^. Gene prediction from the above methods was merged to a consensus gene set using the EVM (v1.1.1)^32^. The functional annotation of the predicted genes of humpback grouper was performed by alignment to the SwissProt^33^, NR^34^, KEGG^35^, Interpro^36^, GO^37^, and Pfam databases^38^. A total of 24,442 protein-coding genes were predicted (Table 4), of which 24, 268 (99.3%) genes were annotated (Fig. 2B). The lengths of average transcript and CDS were 19,080.10 and 1,607.91 bp, respectively (Fig. 2A).

**Table 4:** Summary statistics of predicted protein-coding genes in the assembled genome.

Gene set	Nmuber	Average transcript length (bp)	Average CDS length (bp)	Average exons per gene	Average exon length (bp)	Average intron length (bp)
***De novo***						
Augustus	30,800	13,113.62	1,312.94	7.34	178.81	1,860.59
GlimmerHMM	97,560	9,725.14	606.75	4.26	142.57	2,800.74
SNAP	40,866	34,778.53	1,097.41	7.83	140.20	4933.02
Geneid	31,659	21,781.54	1,369.00	6.80	201.27	3,518.41
Genscan	33,606	22,898.26	1,545.07	8.63	178.96	2,797.26
***Homolog***						
*C. altivelis*	44,047	6,114.79	960.00	4.29	223.62	1,565.37
*E. akaara*	46,506	8,715.41	1,0811.7	5.35	202.13	1,755.44
*E. lanceolatus*	35,787	11,103.76	1,343.05	6.55	294.99	1,758.08
*G. aculeatus*	37,918	8,509.73	1,042.30	5.31	196.38	1,733.56
*L. crocea*	30,196	12,466.57	1,524.71	7.25	210.23	1,749.94
*P. olivaceus*	35,614	10,087.70	1,219.13	6.02	202.57	1,767.19
*S. schlegelii*	35,473	9,460.80	1,236.28	5.96	207.35	1,657.41
**RNA-seq**						
PASA	76,981	15,673.39	1,357.35	8.31	163.26	1,957.31
Cufflinks	66,026	25,660.70	3,700.11	10.38	356.61	2,342.28
EVM	30,917	15,398.43	1,368.41	7.86	174.20	2,046.52

## Data Records

The genome assembly and raw reads of the genome and transcriptome sequencing for humpback grouper were deposited under the Sequence Read Archive SRP322594^39^. The genome assembly was deposited at GenBank with the accession number GCA_019925165.1^40^. Besides, the assembled genome, predicted peptide, CDS, and GO term files were available in the figshare database with the DOI number: 10.6084/m9.figshare.24145230.v2^41^.

## Technical Validation

### Evaluation of the genome assembly and annotation

To evaluate the integrity and accuracy of the genome assembly, the completeness of the final genome assembly was assessed using BUSCO (v4.0)^42^ with the lineage database vertebrata_odb10 and CEGMA (v2.5)^43^. It was shown that the assembly contained 97.3% complete and 1.4% fragmented conserved single copy orthologue genes, and 94.35% of the 248 core eukaryotic genes. By aligning Illumina sequencing reads to the genome using BWA (v0.7.8)^44^, the reads mapping rate and the coverage rates were 99.68% and 99.91%, respectively. It was indicating high mapping efficiency and comprehensive coverage. Thus, all of the above results indicated that we obtained the high-quality genome of humpback grouper.

## Data Availability

No specific code was used in this study. The data analyses used standard bioinformatic tools specified in the methods.
